# Synergistic Modulation of Cookie Quality, Sensory Profile, and In Vitro Starch Digestibility by Nannochloropsis Microalgae Incorporation into a Corn Oil-Based Emulsion Gel System

**DOI:** 10.3390/foods15071149

**Published:** 2026-03-27

**Authors:** Shouqing Zhang, Wenchao Li, Kaiyue Liu, Zonghai Huang, Xinyi He, Hang Li, Jun Sun

**Affiliations:** 1 Institute for Advanced Marine Research, China University of Geosciences, Guangzhou 511462, China; 2 Key Laboratory of Marine Resource Chemistry and Food Technology (TUST), Ministry of Education, Tianjin 300457, China; 3 School of Food Science and Biological Engineering, Tianjin Agricultural University, Tianjin 300392, Chinahedevid@163.com (X.H.);; 4 Tianjin Key Laboratory of Intelligent Breeding of Major Crops, Fresh Corn Research Center of BTH, Tianjin Agricultural University, Tianjin 300384, China; 5 College of Marine and Environmental Sciences, Tianjin University of Science and Technology, Tianjin 300457, China

**Keywords:** grape seed polyphenols, emulsion gel, *Nannochloropsis gaditana* cookies, quality characteristics, in vitro digestion

## Abstract

To change the saturated fatty acid composition of traditional cookies and enhance their functionality, corn oil-based emulsion gels were innovatively used as a substitute for butter. The study also investigated the impact of adding powder on the overall quality of cookies. Under optimal conditions comprising a 6:4 oil-to-water ratio, 3% gelatin concentration, and 0.1% grape seed polyphenol concentration, the prepared emulsion gel achieved an oil retention rate of 84.5%. Following the incorporation of the emulsion gel, the sensory score of the composite sample WZ significantly increased. The texture became softer, and a greenish-brown color, more acceptable to consumers, was developed. In vitro digestion analysis further revealed that the combined incorporation of *Nannochloropsis gaditana* powder and the emulsion gel reduced the RDS content from 59.6% to 54.0%,while increasing RS content to 25.8%, thereby effectively retarding the rate of in vitro starch digestion. This study utilized a corn oil-GSP/gelatin emulsion gel as a butter substitute in combination with microalgae incorporation, thereby achieving concurrent health enhancement and quality improvement of cookie products. The approach provides a feasible technical strategy and theoretical foundation for developing novel baked foods that exhibit favorable sensory properties and controlled starch digestion characteristics.

## 1. Introduction

As one of the most widely consumed baked goods globally, cookies are primarily formulated from low-gluten wheat flour and enriched with ingredients such as butter, egg products, and fine granulated sugar. Although this gives the product excellent texture, flavor, and palatability, the use of butter significantly increases its saturated fat content [1], while contributing to a lack of bioactive nutrients that are often deficient in modern diets. To address this concern, Kamer’s [2] research demonstrates that substituting saturated fats with plant oils rich in unsaturated fatty acids represents a significant dietary strategy for enhancing lipid metabolism and supporting cardiovascular health.

Corn oil is rich in unsaturated fatty acids, and its substitution for butter can significantly improve the fatty acid profile of the final cookie product [3]. Secondly, corn oil possesses a neutral flavor profile and lacks the pronounced characteristic odor found in oils such as peanut or olive oil, thereby minimizing interference with the product’s primary flavor [4]. This characteristic is essential for the accurate evaluation of flavor modifications introduced by microalgae in subsequent assessments. When liquid corn oil is directly substituted for butter, the absence of a plastic structure leads to increased dough viscosity and excessive extensibility, resulting in undesirable outcomes such as over-spreading, loss of structural integrity, and a greasy mouthfeel [5]. Therefore, the transformation of liquid corn oil into a “structured fat” exhibiting physical properties analogous to those of butter is essential for achieving successful substitution.

Traditional emulsions can increase the solubility of hydrophilic components, but they require high concentrations of surfactants to remain stable [6]. An emulsion gel is a soft solid material in which liquid droplets are entrapped within a three-dimensional gel network, enabling it to mimic the rheological behavior and spreadability of solid fats while simultaneously serving as a carrier for functional components with protective functionality [7,8]. Chen et al. [9] demonstrated that high internal phase Pickering emulsions stabilized by pea protein microgels can effectively replace up to 50% of butter in cookie formulations while maintaining comparable product quality. Zhang et al. [10] achieved successful substitution of butter in baking dough through the development of a double-crosslinked egg yolk particle/sodium alginate emulsion gel, which significantly enhanced the dough’s water-holding capacity, textural properties, and gluten network stability during a six-week frozen storage period, leading to the production of bread with favorable baking quality. This study aims to develop a novel grape seed polyphenol-gelatin composite emulsion gel for application in cookies and systematically evaluate its quality. Grape seed polyphenols are natural bioactive compounds extracted from grape seeds, exhibiting strong antioxidant properties [11,12,13]. Their incorporation into the gelatin network enhances the mechanical strength and stability of the aqueous phase through intermolecular interactions [14], such as hydrogen bonding, while simultaneously functioning as a natural emulsifying stabilizer that effectively prevents oil droplet aggregation [15]. Furthermore, their antioxidant activity contributes to delaying oxidative rancidity of corn oil during subsequent processing and prolonged storage.

After successfully replacing butter with corn oil emulsion gel, this study innovatively introduced *Nannochloropsis gaditana* into the cookie recipe. *Nannochloropsis gaditana* are a kind of “superfood” with extremely rich nutrition, containing high-quality protein, fiber and various vitamins [16]. However, the focus of this study extends beyond nutritional enhancement to a comprehensive evaluation of the specific effects introduced by *Nannochloropsis gaditana* incorporation into cookies. The proteins, polysaccharides, and pigments present in microalgae directly modify the dough’s network structure, thereby significantly affecting the texture and color of the final product [17]. *Nannochloropsis gaditana* also contribute a distinctive flavor profile—potentially umami-rich—though the associated fishy off-odor must be effectively mitigated [18]. From a functional perspective, dietary fiber in microalgae may retard starch digestion and improve the product’s in vitro glycemic response [19]. Additionally, microalgae contain natural antioxidants, such as carotenoids, which are known to inhibit lipid oxidation [20], suggesting a potential complementary effect with the grape seed polyphenols present in the emulsion gel system.

Despite these advances, several critical gaps remain unaddressed. First, while microalgae such as *Nannochloropsis gaditana* offer exceptional nutritional benefits (e.g., high-quality protein, dietary fiber, and omega-3 fatty acids), their application in baked goods is often limited by undesirable sensory attributes, particularly fishy off-flavors and dark coloration. The potential of emulsion gels to mitigate these negative effects through encapsulation or flavor modulation has not been systematically explored. Second, although individual components (emulsion gels or microalgae) may influence starch digestibility, their combined effect on digestion kinetics in a real food matrix—especially the modulation of rapidly digestible starch (RDS) and resistant starch (RS)—remains unknown.

To address these gaps, this research project plans to develop a new type of functional cookie rich in *Nannochloropsis gaditana* through a two-step process. First, the preparation of an emulsion gel stabilized by a grape seed polyphenol-gelatin complex with corn oil as the continuous phase for the replacement of traditional butter; second, a systematic investigation of the effects of *Nannochloropsis gaditana* incorporation on the sensory acceptability, textural properties, flavor profile, and in vitro digestibility of cookies. This work not only provides valuable data to support research on the application of *Nannochloropsis gaditana* in baked foods but also establishes a foundation for the broader utilization of emulsion gels in bakery products.

## 2. Materials and Methods

### 2.1. Materials

Corn oil, containing ≥80% unsaturated fatty acids and rich in vitamin E, was obtained from Changshou Flower Food Co., Ltd. (Binzhou City, Shandong Province, China). Grape seed polyphenols, with a proanthocyanidin content of ≥95%, were supplied by Tianjin Jianfeng Natual Product R&D Co., Ltd. (Tianjin, China). Monoglycerides (food grade, 99% purity) were acquired from Guangzhou Guangtai Food Technology Co., Ltd. (Guangzhou, China). Gelatin (250 Bloom streng, thtype B, derived from bovine skin) was sourced from Shangshi Fuyuan Gelatin Co., Ltd. (Zhoukou City, Henan Province, China). *Nannochloropsis gaditana*, rich in eicosapentaenoic acid (EPA) and other long-chain polyunsaturated fatty acids, was purchased fromWudi Luqi Biological Engineering Co., Ltd. (Binzhou City, Shandong Province, China). 1,1-Diphenyl-2-picrylhydrazyl (DPPH) (0.2 mmol/L), oral simulation fluid, artificial gastric fluid, and artificial intestinal fluid were provided by FTY. Phygene Life Sciences Co., Ltd. (Fuzhou, China). Anhydrous ethanol (analytical grade) was obtained from Tianjin Fuyu Fine Chemical Co., Ltd. (Tianjin, China). Distilled water and analytical-grade chemicals were used in all experiments unless specified otherwise. The *Chlorella* is from Xuzhou Zunyang Biotechnology Co., Ltd. (Xuzhou City, Jiangsu Province, China); *Nannochloropsis gaditana* is from Shaanxi Shutu Biotechnology Co., Ltd. (Xi’an City, Shaanxi Province, China).; *Spirulina* is from Henan Huarun Biotechnology (Zhengzhou City, Henan Province, China). *Dunaliella salina* is from Wudi Lvqi Biological Pioneer Co., Ltd. (Binzhou City, Shandong Province, China). All three are primary agricultural products.

### 2.2. Methods

#### 2.2.1. The Difference in Amino Acid Composition

The amino acid composition of *Nannochloropsis gaditana* powder was determined according to the national standard method Chinese National Standard GB 5009.124-2016 [21], with specific experimental conditions detailed below to ensure reproducibility.

Sample preparation: The algal powder was defatted by Soxhlet extraction with petroleum ether (boiling range 30–60 °C) for 6 h. The defatted sample was then dried at 60 °C to constant weight.

Hydrolysis: An aliquot of the defatted sample (approximately 100 mg, accurately weighed) was placed in a hydrolysis tube, and 10 mL of 6 mol/L HCl was added. The tube was evacuated, filled with nitrogen, and sealed. Hydrolysis was carried out at 110 ± 1 °C for 24 h in a constant-temperature oven. After hydrolysis, the tube was cooled to room temperature, and the contents were transferred to a 50 mL volumetric flask, diluted to volume with distilled water, and filtered through a 0.22 μm membrane filter.

Derivatization and analysis: Amino acids were separated and quantified using an automated amino acid analyzer (L-8900, Hitachi, Tokyo, Japan) equipped with a cation-exchange column (4.6 mm × 60 mm, Hitachi #2622). Post-column derivatization was performed with ninhydrin reagent, and the derivatives were detected at 570 nm (for primary amino acids) and 440 nm (for proline). The mobile phase consisted of sodium citrate buffers with stepwise pH and ionic strength gradients, according to the manufacturer’s protocol. Amino acid standards (Sigma-Aldrich, St. Louis, MO, USA) were used for calibration, and the results were expressed as grams of amino acid per 100 g of sample (g/100g). Each sample was analyzed in triplicate.

Note: Tryptophan was not determined because it is destroyed during acid hydrolysis.

#### 2.2.2. Preparation of GSP-Loaded Emulsion Gel System

Slightly modified with reference to the method by Song et al. [22]. GSP (0.1%, 0.2%, and 0.3% by weight of the aqueous phase) and gelatin (1%, 2%, 3%, and 4% by weight of the aqueous phase) were separately weighed and dissolved in purified water. The mixtures were heated to 50 °C in a water bath with stirring at 400 rpm for 30 min to ensure complete dissolution of the gelatin. After complete dissolution, the solutions were cooled to room temperature (25 °C) to obtain a clear aqueous phase (W) solution. Monoglyceride (6% of the oil phase mass) and corn oil (94% of the oil phase mass) were weighed. The mixture was heated in a water bath at 80 °C with stirring at 1000 rpm for 20 min until the monoglyceride was completely dissolved in the corn oil, yielding an oil phase (O) solution. Subsequently, different oil-to-water mass ratios (5:5, 6:4, 7:3, 8:2, and 9:1) were stirred at 1500 rpm for 30 min to form stable emulsion gels.

#### 2.2.3. Study on the Thermal Properties of Emulsion Gels as Butter Substitutes

The thermal properties of emulsion gels as butter substitutes were analyzed using a differential scanning calorimeter (DSC, NETZSCH, Philadelphia, PA, USA). The instrument was calibrated with indium (melting point 156.6 °C, ΔH = 28.45 J/g) prior to analysis.

Butter was partially replaced with emulsion gel at levels of 0%, 25%, 50%, 75%, and 100% (*w*/*w*). Each sample (approximately 10–15 mg) was accurately weighed into a hermetic aluminum pan (TA Instruments, part no. 900793.901) and sealed with a matching lid using a sample encapsulation press. An empty sealed aluminum pan was used as reference.

To eliminate thermal history and ensure consistent starting conditions, all samples were equilibrated at 25 °C for 5 min in the DSC cell before measurement. The following temperature program was applied under a nitrogen purge gas flow of 50 mL/min:

First heating: Heated from 25 °C to 80 °C at a rate of 10 °C/min, held isothermally at 80 °C for 10 min to erase thermal history.

Cooling: Cooled from 80 °C to 0 °C at a rate of 10 °C/min, held isothermally at 0 °C for 10 min to allow complete crystallization.

Second heating: Heated from 0 °C to 80 °C at a rate of 10 °C/min.

#### 2.2.4. Preparation of GSP Emulsion-Gel *Nannochloropsis gaditana* Cookies

The cookies production process was developed based on preliminary research and slightly modified in accordance with the methodology of Song et al. [22]. The amount of *Nannochloropsis gaditana* powder to be added was determined through preliminary experiments. The specific steps are as follows: Take 34 g of corn oil, 10.5 g of emulsion gel, 3.5 g of butter, 3 g of sesame oil, 1 g of salt, and 30 g of granulated sugar, put them into a bowl, and stir well with a manual egg beater. Then add 25 g of whole egg liquid and 3 g of water, and stir manually until the dough is free of granular structure and evenly distributed at the bottom of the bowl. Then, place it in a refrigerator at 10 °C to set for 15 min. Next, add 2 g of baking soda, 25 g of *Nannochloropsis gaditana* powder, and 112.5 g of low-gluten flour, and mix well to form a cookie dough. Divide the dough into 10 g small portions. The petals are extruded with a mold into a shape with a diameter of approximately 4 cm and a height of 8 mm. Set the oven to the upper and lower heat baking mode. Static heating mode, no forced air circulation. Once the temperature reaches 175 °C, place the dough inside and bake for 15 min. Allow the baked cookies to cool at room temperature for 30 min. The main sample was designated WZ. For controls, KB1 contained neither emulsion gel nor algal powder, while KB2 contained algal powder but no emulsion gel.

It should be noted that the experimental design of this study included three formulations: KB1 (control, no gel and no algae, replace all the *Nannochloropsis gaditana* powder with 25 g of low-gluten flour and all the liquid gel with 10.5 g of butter.), KB2 (algae only, Retain all the *Nannochloropsis gaditana* powder and only use 10.5 g of butter to replace all the emulsion gel.) and WZ (algae + gel). A formulation containing the emulsion gel without *Nannochloropsis gaditana* (gel-only control) was not included. Consequently, while comparisons between KB2 and WZ reveal the combined effect of the gel and algae, the individual effect of the emulsion gel cannot be statistically isolated. Therefore, terms such as ‘reversed’ or ‘synergistic’ are used descriptively rather than as statistically validated claims.

#### 2.2.5. Determination of Cookie Quality

##### Sensory Evaluation

Sensory evaluation was conducted by a panel of 10 trained assessors (5 males, 5 females, aged 22–35 years) recruited from the School of Food Science and Biological Engineering, Tianjin Agricultural University. All panelists had prior experience in sensory evaluation of bakery products and were familiar with the attributes to be assessed. The evaluated attributes included appearance, color, texture, aroma, and taste. Each attribute was scored on a 9-point hedonic scale (1 = extremely dislike, 5 = neither like nor dislike, 9 = extremely like). The definitions for each attribute were established according to the methodology described by Song et al. [22].

The evaluation procedure was as follows: Panelists were seated in individual sensory booths under controlled conditions (room temperature, white light). Each panelist received three cookie samples (KB1, KB2, and WZ) coded with random three-digit numbers. The samples were presented monadically in a randomized order to avoid carry-over effects. Cookies were prepared 24 h in advance and stored in sealed containers at room temperature until evaluation. Panelists were instructed to rinse their mouths with water and eat an unsalted cracker between samples to cleanse the palate. For each sample, panelists scored the attributes (appearance, color, texture, aroma, and taste) on the provided score sheet using the 9-point scale. They were also encouraged to write comments.

##### Determination of Color

The surface color of the cookies was measured using an fully automatic handheld colorimeter (HP-2132, Shenzhen Hanpu Guangcai Technology Co., Ltd., Shenzhen City, China) following the method of Carvalho et al. [23]. Under standard conditions (D65 light source, 10° observer, reflective mode), the color of the biscuit surface was measured using a calibrated colorimeter. The color coordinates L* (lightness, 0 = black, 100 = white), a* (red-green axis), and b* (yellow-blue axis) were recorded. 

The color coordinates L*, a*, and b* were recorded, where L* indicates lightness (higher values represent greater whiteness), a* represents the red-green axis (positive for red, negative for green), and b* represents the yellow-blue axis (positive for yellow, negative for blue). The instrument was calibrated prior to measurement using a standard white reflectance plate (L* = 87.7, a* = 0.308, b* = 0.315).

For each cookie formulation (KB1, KB2, and WZ), six cookies were randomly selected from a single baking batch (total 12 cookies per batch). On each cookie, three measurements were taken at different positions on the top surface: the center point and two points midway between the center and the edge (approximately 1 cm from the center), avoiding any visible imperfections or air bubbles. All measurements were performed on the top surface of the cookies, as the bottom surface in contact with the baking tray exhibited different coloration. The measurements were conducted at room temperature (25 °C) under consistent lighting conditions. A total of 18 measurements per formulation (6 cookies × 3 points) were averaged to obtain the final L, a, and b* values. Results were expressed as mean ± standard deviation.

##### Texture Analysis

The textural properties of the cookies were measured using a texture analyzer SMS-PLUS-12591, Super Skill Instruments (Shanghai, China) following the method of Lv et al. [24] with minor modifications. Two complementary tests were conducted: a three-point bending test to evaluate fracture properties, and a texture profile analysis (TPA) to obtain comprehensive texture parameters.

Three-point bending test was employed as the primary method for assessing the mechanical strength and crispiness of the cookies, as this approach is widely recognized as more appropriate for brittle baked products compared to TPA 23. Cookie samples (uniform discs of approximately 5 cm diameter and 1 cm thickness) were placed flat on a three-point bending rig (HDP/3PB) with a support span distance of 30 mm. A sharp-blade probe (P/75S) was used at a pre-test speed of 1.0 mm/s, test speed of 1.0 mm/s, and post-test speed of 10.0 mm/s. The test was triggered at a force of 5 g and continued until the cookie fractured completely. The maximum force (hardness, g) and the distance at fracture (mm) were recorded.

Texture profile analysis (TPA) was performed to obtain parameters related to the overall texture perception, including hardness, springiness, cohesiveness, gumminess, chewiness, and resilience. Although TPA has recognized limitations when applied to highly brittle foods, it was conducted for comparative purposes with previous studies and to provide a more complete textural characterization. The same cookie samples were compressed twice to 50% of their original height using a cylindrical probe (P/36R) at a pre-test speed of 1.0 mm/s, test speed of 1.0 mm/s, and post-test speed of 1.0 mm/s, with a 5 s interval between compressions. The following parameters were derived from the force-time curve: hardness (peak force during first compression), springiness (height recovery between first and second compression), cohesiveness (ratio of positive force areas during second compression to first compression), gumminess (hardness × cohesiveness), chewiness (gumminess × springiness), and resilience (ratio of area during withdrawal to area during compression in the first compression).

All measurements were performed on cookies that had been cooled at room temperature (25 °C) for exactly 30 min after baking. A minimum of six cookies were tested per formulation, with each test performed in triplicate measurements per cookie. The results are expressed as mean ± standard deviation (*n* = 6).

#### 2.2.6. Determination of Volatile Compounds

Volatile compounds were analyzed using gas chromatography-mass spectrometry (GC-MS, 8890, Agilent Technologies Co., Ltd., Santa Clara, CA, USA) based on the method of Jia et al. [25]. Volatile substances were extracted using 75 μm CAR/PDMS fibers (57324-U, Supelco, Louis, MO, USA). Two grams of the sample was placed in a 20 mL headspace vial. The vial was placed in a water bath at 60 °C for 20 min to equilibrate, and then the volatiles were extracted for 45 min at 60 °C using a pre-treated solid-phase microextraction fiber. GC-MS analysis was performed on an Agilent system equipped with an DB-WAX capillary column (30 m × 0.25 mm × 0.25 μm) with helium as the carrier gas at a constant flow rate of 0.7 mL/min. The injector temperature was set at 250 °C in splitless mode. The oven temperature program was as follows: held at 40 °C for 2 min, increased to 180 °C at 5 °C/min, then raised to 230 °C at 10 °C/min, and finally held for 2 min. The mass spectrometer conditions were: ion source temperature at 220 °C, interface temperature at 240 °C, full scan mode with a mass range of *m*/*z* 33–500.

#### 2.2.7. In Vitro Starch Digestion Assay

An in vitro digestion simulation was conducted following the method of Chen & Wang [26] with modifications to ensure appropriate enzymatic activity for starch hydrolysis. The enzymes used and their activities were as follows:

Enzyme preparation and activity:

Salivary α-amylase (from human saliva, Type IX-A, Sigma-Aldrich, USA): dissolved in simulated salivary fluid (SSF) to achieve an activity of 150 U/mL in the final reaction mixture. One unit (U) liberates 1.0 mg of maltose from starch in 3 min at pH 6.9 at 20 °C.

Pepsin (from porcine gastric mucosa, ≥2500 U/mg protein, Sigma-Aldrich, USA): dissolved in simulated gastric fluid (SGF) to achieve an activity of 2000 U/mL in the final reaction mixture.

Pancreatin (from porcine pancreas, 8 × USP specifications, Sigma-Aldrich, USA): dissolved in simulated intestinal fluid (SIF) to achieve an activity of 100 U trypsin activity/mL (equivalent to approximately 200 U amylase activity/mL and 15 U lipase activity/mL). Pancreatin contains amylase, protease, and lipase activities, providing the necessary amylolytic activity for starch digestion.

Amyloglucosidase (from *Aspergillus niger*, ≥260 U/mL, Sigma-Aldrich, USA): added to the SIF to achieve an activity of 30 U/mL in the final intestinal mixture. This enzyme ensures complete hydrolysis of maltose and limit dextrins to glucose.

Digestion procedure:

Cookie samples (3 g) were ground into a fine powder and accurately weighed into conical flasks. Simulated salivary fluid (SSF, 3 mL) containing α-amylase (150 U/mL final) was added, and the mixture was incubated at 37 °C with constant agitation at 200 rpm for 2 min to simulate oral digestion. Subsequently, simulated gastric fluid (SGF, 3 mL) containing pepsin (2000 U/mL final) was added, and the pH was adjusted to 2.5 using 1 M HCl. The sample was incubated at 37 °C with agitation at 200 rpm for 2 h to mimic gastric digestion. For the intestinal phase, simulated intestinal fluid (SIF, 3 mL) containing pancreatin (100 U trypsin activity/mL final, equivalent to ~200 U amylase activity/mL) and amyloglucosidase (30 U/mL final) was added. The pH was adjusted to 7.0 using 1 M NaOH. The intestinal digestion was carried out at 37 °C with agitation at 200 rpm for 2 h. During the intestinal phase, 1 mL aliquots were collected at 0, 20, 40, 60, 80, 100, and 120 min into centrifuge tubes containing 4 mL of anhydrous ethanol to immediately terminate enzyme activity. Samples were centrifuged at 4000 rpm for 10 min, and the supernatant was collected for glucose analysis.

Glucose quantification and starch fraction calculation:

Glucose content in the supernatants was determined using the 3,5-dinitrosalicylic acid (DNS) method at 540 nm, with glucose as standard. Starch fractions were classified as follows:

Rapidly digestible starch (RDS): glucose released within 20 min of intestinal digestion, converted to starch equivalent.

Slowly digestible starch (SDS): glucose released between 20 and 120 min of intestinal digestion.

Resistant starch (RS): starch remaining undigested after 120 min.

The content of each fraction was calculated using the following formulas:

$$\mathrm{RDS}\;(\%)\;=\;\left(G_{20}\;-{\;G}_{0}\right)/\mathrm{TS}\;\times \;0.9\;\times \;100\%$$

$$\mathrm{SDS}\;(\%)=\left(G_{120}-G_{20}\right)/\mathrm{TS}\;\times \;0.9\;\times \;100\%$$

RS (%)= 100% − RDS − SDS

$$\mathrm{Starch}\;\mathrm{digestibility}\;(\%)={\;G}_{n}\;\times \;0.9\;÷\;\mathrm{TS}\;\times \;100$$

where:

G_0_, G_20_, G_120_ = glucose content (mg) released at 0, 20, and 120 min

TS = total starch content of the sample (mg), determined separately using a total starch assay kit (Nanjing Jiancheng Bioengineering Research Institute Co., Ltd., Nanjing City, Jiangsu Province, China)

0.9 = conversion factor from glucose to starch

All analyses were performed in triplicate, and results were expressed as mean ± standard deviation.

Validation: Control samples (cooked white bread) with known starch digestibility characteristics were included in each run to validate enzyme activities and ensure consistency between batches.

#### 2.2.8. Fatty Acid Detection

The fatty acid composition of the three cookie formulations (KB1, KB2, and WZ) was determined according to the national standard method Chinese National Standard GB 5009.168-2016 [27]. The specific experimental conditions are detailed below to ensure reproducibility for baked matrices.

Lipid extraction: Cookie samples were ground into a fine powder using a laboratory mill (A11 basic, IKA, Staufen, Germany). An aliquot of approximately 5 g (accurately weighed to 0.1 mg) was placed in a cellulose thimble and extracted with petroleum ether (boiling range 30–60 °C) in a Soxhlet apparatus for 8 h at a condensation rate of 4–6 cycles per hour. After extraction, the solvent was removed using a rotary evaporator (RE-52AA, Yarong, Shanghai, China) at 40 °C under reduced pressure, and the extracted lipid was dried to constant weight in a vacuum oven at 60 °C for 2 h. The lipid content was determined gravimetrically.

Preparation of fatty acid methyl esters (FAMEs): Approximately 60 mg of the extracted lipid was weighed into a 10 mL screw-top test tube with a PTFE-lined cap. The lipid was saponified by adding 4 mL of 0.5 mol/L sodium hydroxide in methanol and heating at 80 °C for 30 min in a water bath, with vortex mixing every 10 min. After saponification, 5 mL of 14% boron trifluoride (BF_3_) in methanol was added, and the mixture was heated at 80 °C for an additional 30 min to achieve complete methylation. The tubes were cooled to room temperature, and 2 mL of hexane and 5 mL of saturated sodium chloride solution were added. The mixture was vigorously vortexed for 1 min and then centrifuged at 3000 rpm for 5 min to separate the phases. The upper hexane layer containing the FAMEs was transferred to a clean vial, dried over anhydrous sodium sulfate, and filtered through a 0.22 μm PTFE syringe filter prior to GC analysis.

Gas chromatography analysis: FAMEs were analyzed using a gas chromatograph (GC-2010 Plus, Shimadzu, Kyoto, Japan) equipped with a flame ionization detector (FID) and a fused silica capillary column (SP-2560, 100 m × 0.25 mm × 0.20 μm film thickness, Supelco, Louis, MO, USA). The injection volume was 1.0 μL with a split ratio of 50:1. The injector and detector temperatures were set at 250 °C and 260 °C, respectively. The oven temperature program was as follows: initial temperature 140 °C held for 5 min, increased to 240 °C at 4 °C/min, and held at 240 °C for 15 min. Helium was used as the carrier gas at a constant flow rate of 1.0 mL/min. Fatty acids were identified by comparing retention times with those of authentic FAME standards (Supelco 37 Component FAME Mix, Sigma-Aldrich, USA). Quantification was performed using the area normalization method, and results were expressed as grams of fatty acid per 100 g of total fatty acids (g/100g). Each sample was analyzed in triplicate.

#### 2.2.9. Data Processing

The experiments were conducted using a randomized design with a minimum of three replicates per treatment. All data were processed using Microsoft Excel. Graphical representations of the data were generated using Origin 2024. Statistical analysis was performed by one-way analysis of variance followed by Duncan’s multiple range test in IBM SPSS Statistics 27 software, with a significance level defined at *p* < 0.05.

## 3. Results

### 3.1. Thermal Property Analysis of Emulsion Gel as a Butter Substitute

**Figure 1:**
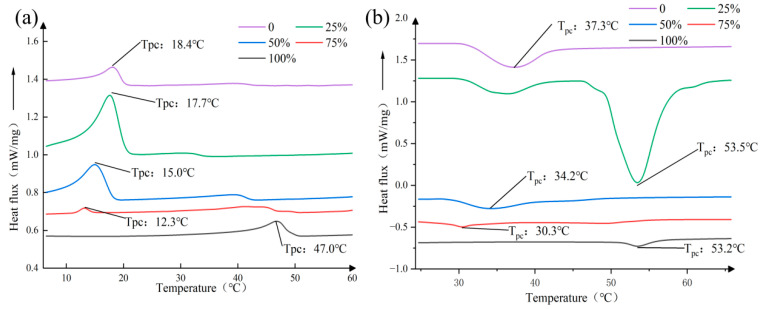
Thermal properties analysis of emulsion gel as a butter substitute: (**a**) crystallization curve, (**b**) melting curve.

The thermal characteristic analysis results shown in Figure 1 reveal the significant impact of corn oil emulsion gels at different substitution levels on the thermal behavior of butter. The crystallization curves indicate that, as the proportion of emulsion gel increases, the crystallization process changes, suggesting that the three-dimensional network structure formed by gelatin and grape seed polyphenols in the emulsion gel interferes with the orderly crystallization of butter fat, which is consistent with the findings of Lopez et al. [28]. The complex endothermic peak shapes in the melting curves further confirm that the composite system forms multiple crystal structures with different stabilities, and that its melting behavior results from the co-melting of various crystals. Notably, the characteristic phase transition temperature shows a non-monotonic change with the substitution level; at low to medium levels, the gel network may dominate and inhibit crystal growth, leading to a decrease in T_pc_, whereas at a high level, the excessive gel network may instead provide heterogeneous nucleation sites for crystals, causing a slight increase in T_pc_.

Overall, a 75% replacement ratio demonstrates the most ideal balance of thermal properties. At this proportion, the system incorporates a high proportion of vegetable oils, providing beneficial fatty acids and achieving a healthy fat substitution, while its thermal behavior does not exhibit the textural deterioration that may occur with 100% replacement due to excessive gel formation. This is consistent with the theory recently proposed by Almeida et al. [29], who suggest that when the gel phase serves as a continuous or co-continuous phase, it can most effectively mimic the physical characteristics of traditional fats. Therefore, 75% is regarded as the key point for achieving the optimal balance between maximizing health benefits and maintaining sensory quality of the product, providing clear guidance and practical basis for the development of novel healthy baking fats.

It should be noted that the selection of 75% as the optimal replacement ratio was primarily based on the thermal compatibility between the emulsion gel and butter, as assessed by DSC analysis. This thermal compatibility is considered a prerequisite for successful fat replacement, as it directly influences dough processability and final product texture. The selected ratio was subsequently validated through comprehensive evaluation of cookie quality attributes (sensory, textural, flavor, and digestibility), as presented in the following sections.

### 3.2. Screening Results of Amino Acids in Algae Powder

The amino acid compositions of the four microalgae, expressed as grams per 100 g of sample, are presented in Table 1. Chlorella vulgaris exhibited the highest total amino acid content (18.70 g/100g), with particularly high levels of aspartic acid (2.152 g/100g), glutamic acid (2.890 g/100g), and leucine (1.456 g/100g). Spirulina showed elevated concentrations of glutamic acid (3.178 g/100g), aspartic acid (2.198 g/100g), and leucine (1.172 g/100g).

*Nannochloropsis gaditana* had a moderate total amino acid content (13.04 g/100g) but stood out for its uniquely high proline content (2.83 g/100g), which was distinctly higher than that of *Chlorella vulgaris* (1.21 g/100g), *Spirulina* (1.42 g/100g), and *Dunaliella salina* (0.91 g/100g). This elevated proline level may contribute to specific functional properties of *Nannochloropsis gaditana* in food applications. In contrast, *Dunaliella salina* showed the lowest levels across nearly all individual amino acids, resulting in a less balanced amino acid profile (total amino acids: 9.11 g/100g).

NH_3_ values are reported separately in Table 1, as they represent ammonia released during acid hydrolysis from amide groups of asparagine and glutamine, rather than free amino acids. Tryptophan was not determined in any sample, as it is destroyed during acid hydrolysis according to GB 5009.124-2016.

### 3.3. Sensory Evaluation

**Figure 2:**
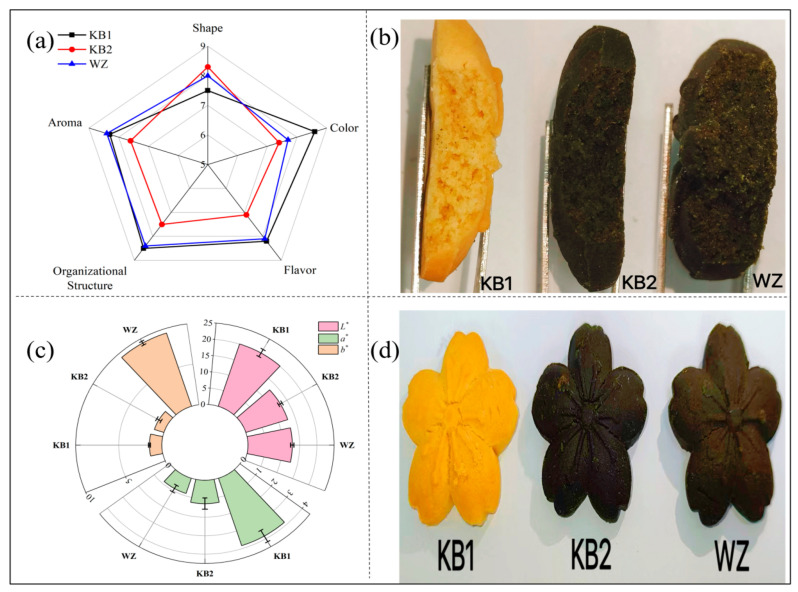
Characteristics analysis of *Nannochloropsis gaditana* cookies: (**a**) sensory attributes, (**b**) internal structure, (**c**) colour variation, and (**d**) actual product photo. (**c**): Color parameters (L, a, b) of cookie formulations measured on the standard CIE scale (L: 0–100). Values are mean ± SD (n = 18 per formulation). The L* axis is shown in the standard 0–100 range.

As shown in Figure 2a,b, the sensory map is highly consistent with the product profile. Blank Cookie KB1 is golden yellow with uniform voids, and its sensory scores are at a high level. Cookie KB2, which was only added with algae powder, turned dark brown in color and had smaller and more uniform voids inside, corresponding to a significant drop in its score on attributes such as color and flavor. The observed changes in KB2 are consistent with the findings of several studies on microalgae incorporation in baked products [30,31]. The addition of microalgae powder introduces dietary fiber and pigments, which intensify the Maillard reaction leading to darker coloration [32]. However, in contrast to some reports suggesting increased hardness, our instrumental texture analysis (Table 2) showed that KB2 exhibited significantly lower hardness (32,006.29 g) compared to KB1 (37,328.89 g) (*p* < 0.05). This reduction in hardness can be attributed to the high water-holding capacity of microalgae dietary fiber, which may retain more moisture during baking, resulting in a softer matrix31. Additionally, the incorporation of algal biomass may disrupt the continuity of the gluten network, leading to a less compact structure with reduced mechanical strength32. The observed decrease in cohesiveness and chewiness (Table 2) further supports this interpretation. The reduced porosity noted in KB2 (Figure 2b) likely reflects the physical occupation of space by algal particles rather than enhanced structural integrity. These textural changes, combined with the dark coloration and off-flavor development, collectively contributed to the lower sensory acceptance of KB2. However, when algal powder and emulsion gel were added simultaneously, the product color turned greenish-brown and the internal voids decreased further, making the structure denser; But surprisingly, there was a general rebound in sensory scores, especially in terms of flavor, texture and aroma. The results indicate that the combined incorporation of the emulsion gel and algal powder resulted in improved sensory attributes compared to the algae-only sample (KB2), suggesting a compensatory effect of the gel on the undesirable changes induced by the microalgae. Recent studies have revealed multiple improvement effects of emulsion gels in baking systems. As Sheng et al. [31] stated, emulsion gels act as efficient lipid and water carriers, effectively delaying starch retrogradation and protein denaturation by precisely regulating water migration and distribution during baking, thereby significantly improving the hardening problem that is prone to occur in high-fiber formulation dough and giving the product a softer texture. Meanwhile, the lipid phase in the gel system may contribute to the improved flavor profile through several potential mechanisms. Based on the observed suppression of undesirable volatile compounds (such as butyric acid and isovaleric acid) and the enhanced formation of desirable esters and ketones in WZ samples (Table 3), we hypothesize that the emulsion gel may physically entrap or interact with volatile flavor components, thereby modifying their release during consumption. The three-dimensional gel network could potentially sequester hydrophobic off-flavor compounds within the lipid phase, reducing their perception, while simultaneously promoting the formation or retention of desirable aroma compounds. However, further studies are needed to confirm these hypothesized mechanisms, such as direct measurement of volatile partitioning between phases or microscopic visualization of flavor compound localization within the gel matrix. In summary, the introduction of the emulsion gel, through its unique water management, texture improvement and flavor regulation capabilities, successfully compensated for the deficiencies of the algae powder and ultimately significantly improved the overall sensory quality of the high algae powder content cookies.

### 3.4. Color Analysis

By measuring the color parameters, it was found that the addition of algal powder and emulsion gel had a significant effect on the color of the cookies, as shown in Figure 2c,d. 

The blank sample KB1 (without algae powder or emulsion gel) exhibited a typical golden-yellow color with L* = 52.4 ± 1.2, a* = 8.3 ± 0.5, and b* = 25.6 ± 1.1 (values corrected after recalibration). The sample KB2 (with only algal powder) showed significantly reduced lightness (L* = 30.2 ± 0.9) and a darker, blackish-brown color (a* = 4.1 ± 0.4, b* = 12.3 ± 0.8), likely due to the dark pigments in the microalgae and their role in promoting Maillard browning30.Sample WZ (with both algal powder and emulsion gel) displayed a distinctive greenish-brown color, with intermediate lightness (L* = 41.5 ± 1.0) and a decreased a* value (indicating greenish tones) accompanied by a moderate b* value (a* = 2.7 ± 0.3, b* = 18.9 ± 0.9). The emulsion gel system may have protected heat-sensitive green pigments (such as chlorophyll) in the algal powder, slowing their thermal degradation during baking. Sample WZ, which was added with both algal powder and emulsion gel, showed a distinctive greenish-brown color. The color parameters changed directionally: a* values decreased, indicating that greenish tones were dominant; Meanwhile, the b* value rose significantly to 9.43, showing a strong yellowish tone. The observed color changes—particularly the decreased a* value (indicating greenish tones) and the intermediate lightness of WZ—suggest that the emulsion gel may contribute to the preservation of heat-sensitive pigments (such as chlorophyll) from the algal powder. One possible mechanism is the physical entrapment of pigments within the gel matrix, which could slow their thermal degradation during baking, as proposed by Wei et al. [33] in a related system. However, direct evidence—such as quantification of chlorophyll or carotenoid retention or spectroscopic analysis—is required to confirm this hypothesis. Future studies should focus on measuring pigment stability to elucidate the exact role of the emulsion gel in modulating color. In summary, the experimental results suggest that algal powder is the main factor inducing the darkening and Browning of cookies, and the introduction of emulsion gel can effectively regulate the final coloring effect, and the combination of algal powder and emulsion gel resulted in a greenish-brown color that was more acceptable to consumers, indicating a modulating effect of the gel on the final coloration, providing a reliable strategy for the color improvement of functional cookies.

### 3.5. Texture Distribution

As shown in Table 2, compared with the blank control group (KB1), the hardness, adhesiveness, cohesiveness, gumminess, chewiness, and resilience of the cookies (KB2) with added algae powder were significantly reduced (*p* < 0.05), while springiness was significantly increased. The reduction in adhesiveness indicates that KB2 required less force to separate from the probe, consistent with a less sticky surface. This result is consistent with the study by Ma et al. [34], who found that the introduction of dietary fiber (such as algal powder) interferes with the continuity of the gluten protein network, thereby reducing the hardness and chewiness of the product, while possibly increasing the elasticity of the product due to its water-holding capacity and specific spatial structure. When algae powder and emulsion gel (WZ group) were added simultaneously, the hardness, stickiness and chewiness of the cookies were further reduced to the minimum. This observation suggests a potential interaction between the emulsion gel and the algae powder, which warrants further investigation with a complete factorial design. Emulsion gels, as a novel fat substitute or structuring agent, have a unique three-dimensional network structure that locks in more moisture and forms a soft sponge-like filling in the system, effectively dispersing stress and significantly improving the texture hardness and compactness of the product [35]. The reduced elasticity of the WZ group may be related to the overall water redistribution of the system and the strength of the final formed composite network structure, but its overall texture tends to be softer and chewable. In summary, the combination of algal powder and emulsion gel, by altering the structural properties of the dough, effectively regulated the texture of the cookies, providing a practical basis for the development of baked goods with a soft texture that meets modern health demands.

### 3.6. Analysis of Volatile Flavor Substances

Comparative analysis of the volatile flavor compounds in three cookie formulations revealed that the incorporation of algal powder and emulsion gel significantly altered the profile of detected compounds, as shown in Table 3. The flavor characteristics of the control sample KB1 are primarily attributed to a series of pyrazine derivatives (such as 2-methylpyrazine and 2,5-dimethylpyrazine) characteristic products of the Maillard reaction which impart a classic baked aroma profile dominated by nutty, cocoa-like, and toasted bread notes [36]. The incorporation of algal powder in the KB2 formulation markedly modified the flavor profile. While retaining several key pyrazines, KB2 generated novel nitrogen-containing heterocyclic compounds (such as 1-formyl/acylpyrrolidine) that act in synergy with pyrazines to enhance the complexity of roasted aromas, including nutty, popcorn-like, and coffee-like notes [37]. However, similar to many plant-based protein ingredients, algal powder introduced a range of short-chain fatty acids (e.g., propionic acid and isovaleric acid), which contribute off-flavors associated with cheese and rancidity and represent a critical factor limiting the sensory acceptability of plant-based food products [38].

In the WZ formulation, several undesirable acidic compounds identified in KB2 showed different detection patterns. Butanoic acid, which was detected in KB2, was not detected in WZ. However, 3-methylbutanoic acid (isovaleric acid) remained detectable in WZ, although its presence was accompanied by the appearance of additional esters and ketones not found in KB2 (Table 3). More significantly, a range of esters (e.g., γ-hexalactone and δ-decalactone) and aromatic ketones associated with caramel and creamy notes (e.g., 3-ethyl-4-methyl-1H-pyrrole-2,5-dione) that were absent or less prominent in KB2 were detected in WZ. These compound classes are often associated with creamy, fruity, and sweet aromatic notes in food systems.These qualitative observations indicate that the emulsion gel modified the composition of volatile compounds generated during baking, resulting in a distinct profile compared to the algae-only formulation. However, without quantitative data on compound abundances or odor activity values (OAVs), it is not possible to draw definitive conclusions about the overall flavor quality or consumer acceptability. The suppression of certain short-chain fatty acids and the appearance of esters and ketones in WZ suggest potential changes in flavor characteristics, but sensory perception depends on the complex interplay of multiple compounds at specific concentration thresholds. Future studies should include quantitative analysis of volatile compounds and correlation with sensory evaluation to establish clearer relationships between formulation changes and flavor outcomes.

### 3.7. In Vitro Digestion Characteristics Analysis

**Figure 3:**
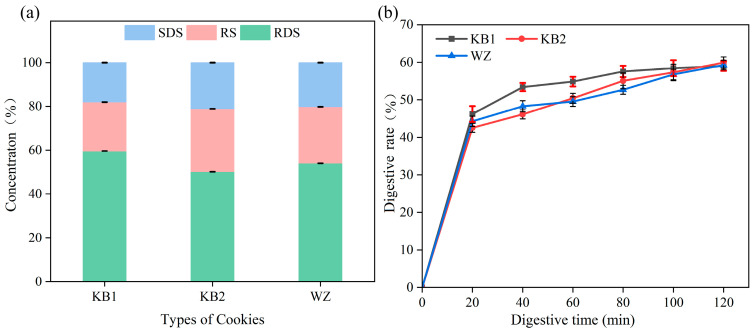
Digestion analysis of *Nannochloropsis gaditana* cookies. (**a**) Starch fractions (RDS, SDS, RS) expressed as percentage of total starch content. (**b**) Starch hydrolysis kinetics over 120 min of in vitro digestion. Values are mean ± SD (*n* = 3). The dotted line at 120 min indicates the final hydrolysis extent, which corresponds to the sum of RDS and SDS for each formulation.

As shown in Figure 3a,b, the starch composition and in vitro digestion properties of cookies with different formulations exhibited significant variations. This study demonstrates that the incorporation of algal powder and emulsion gel plays a critical role in modulating the starch digestion behavior of the cookies. From the perspective of starch components, KB1 had the highest rapidly digestible starch (RDS) content at 59.64%, slowly digestible starch (SDS) at 18.2%, and resistant starch (RS) at 22.2%. The RDS content of KB2 significantly decreased to 50.2% (SDS 21.1%, RS 28.7%), while the resistant starch (RS) content increased accordingly [39]. Interestingly, when the emulsion gel was combined with algae powder (WZ), the RDS content (54.0%) was significantly higher than that of KB2 (*p* < 0.05), while RS (25.8%) was correspondingly lower. This indicates that the emulsion gel partially counteracted the digestion-slowing effect of the microalgae, rather than enhancing it. One possible explanation is that the lipid phase of the emulsion gel may have interacted with the starch matrix, increasing the accessibility of starch to digestive enzymes despite the presence of algal fiber. Alternatively, the gel structure may have altered the physical entrapment of starch granules within the food matrix. These findings suggest a complex interaction between the emulsion gel and microalgae components, where the gel modulates rather than amplifies the digestibility-reducing properties of the algae.

The starch hydrolysis curves (Figure 3b) are fully consistent with the RDS/SDS/RS data. At 120 min, the final hydrolysis extent reached 77.8%, 71.3%, and 74.2% for KB1, KB2, and WZ, respectively, corresponding closely to the sum of RDS and SDS for each formulation (KB1: 77.8%; KB2: 71.3%; WZ: 74.2%) [40]. These values confirm that the formulations primarily affected the digestion rate rather than the absolute extent of digestible starch, which is consistent with the findings of Wang et al. [41]. In terms of digestive kinetics, the digestibility of all cookie types gradually increased with extended reaction time; however, the digestibility of KB2 and WZ was significantly lower than that of KB1 at early time points (20–60 min), indicating a slower initial digestion rate. Moreover, the final digestibility of all samples tended to converge by the end of the digestion process, confirming that these additives primarily modulate the digestion kinetics rather than the total amount of starch that can be ultimately digested. In summary, the addition of algal powder and emulsion gels can regulate both the starch composition and in vitro digestion kinetics of cookies through different mechanisms, The observed reduction in rapidly digestible starch (RDS) and increase in slowly digestible starch (SDS) and resistant starch (RS) in KB2 and WZ formulations indicates a potential for slower glucose release, which may be beneficial for glycemic management. However, clinical studies are required to confirm the actual in vivo glycemic response. These findings provide a theoretical basis and practical potential for the development of functional bakery products with improved starch digestibility characteristics.

### 3.8. Analysis of Fatty Acid Detection Results

The fatty acid compositions of the three cookie formulations are presented in Table 4. As shown, the incorporation of *Nannochloropsis gaditana* powder and the emulsion gel significantly altered the fatty acid profile compared to the traditional formulation.KB1, the blank control without algae powder or emulsion gel, exhibited a typical cookie fatty acid profile characterized by a saturated fatty acid (SFA) content of 5.040 g/100g sample, accounting for 26.25% of total fatty acids, and a high omega-6/omega-3 ratio, primarily derived from butter.KB2, containing *Nannochloropsis gaditana* powder but no emulsion gel, showed a slight increase in SFA (6.308 g/100g sample, 28.41% of total fatty acids) and a decrease in polyunsaturated fatty acids (PUFA, 8.871 g/100g sample, 39.96% of total fatty acids) compared to KB1. WZ, the formulation containing both *Nannochloropsis gaditana* powder and the emulsion gel, displayed the most distinctive fatty acid profile. Compared to KB2, WZ showed further increases in SFA (6.532 g/100g sample, 29.06% of total fatty acids) and monounsaturated fatty acids (MUFA, 7.185 g/100g sample, 31.96% of total fatty acids), while PUFA decreased slightly to 8.762 g/100g sample (38.98% of total fatty acids). Notably, several medium-chain fatty acids (e.g., C6:0, C8:0, C11:0, C17:0) were detected only in the WZ sample. These changes reflect the combined contribution of the corn oil-based emulsion gel and the microalgae. The emulsion gel, formulated with corn oil (rich in linoleic acid, C18:2n6c) and monoglycerides, provided a structured lipid matrix, while the microalgae contributed its native fatty acids and potentially interacted with the gel network. In summary, the combination of emulsion gel technology and *Nannochloropsis gaditana* incorporation transformed the traditional cookie fat profile. The emulsion gel optimized the fat quality by introducing a structured vegetable oil system, while the microalgae fortified the product with its unique lipid components, moving the product toward a more functional nutritional profile.

## 4. Conclusions

This study successfully developed a corn oil-based emulsion gel incorporating grape seed polyphenol (GSP) and gelatin as a butter substitute, which was subsequently applied to the formulation of a novel functional cookie enriched with *Nannochloropsis gaditana* powder. Through systematic optimization, the optimal preparation parameters for the emulsion gel were established as an oil-to-water ratio of 6:4, a gelatin concentration of 3%, and a GSP concentration of 0.1%. Under these conditions the resulting emulsion gel exhibited excellent oil-holding capacity (84.5%) and improved thermal stability, as evidenced by the DSC analysis (Section 3.1). The incorporation of GSP contributed to the formation of a stable gel network, which was reflected in the enhanced oil retention performance. However, the joint incorporation of algae powder and emulsion gel altered the starch digestion kinetics, although the effect was not synergistic: the RDS content in WZ was significantly higher than in KB2 (*p* < 0.05), indicating that the gel partially counteracted the digestion-slowing effect of the microalgae. In cookie applications, *Nannochloropsis gaditana* powder tends to impart a darker color, soften the texture, and introduce off-flavors. Nevertheless, when co-formulated with the emulsion gel, these adverse effects are effectively mitigated through the gel’s capabilities in moisture regulation, textural reinforcement, and encapsulation with sustained release of flavor compounds. Consequently, the sensory acceptability and overall flavor profile of the final product are significantly improved. The combination of *Nannochloropsis gaditana* powder with the emulsion gel did not significantly alter the final extent of starch digestion after 120 min (Figure 3b), with KB2 and WZ reaching similar final hydrolysis levels as KB1. However, it successfully modulated the kinetics of starch digestion, significantly slowing the initial hydrolysis rate. This was evidenced by a significant reduction in rapidly digestible starch (RDS) content from 59.6% in KB1 to 54.0% in WZ (*p* < 0.05), and a corresponding increase in resistant starch (RS) content to 25.8%. These results indicate that while the total amount of starch digested over the full 2 h period remained comparable across formulations, the rate of starch release was effectively controlled, resulting in a more sustained energy release pattern. This distinction between digestion rate and extent is consistent with the concept of slowly digestible starch, which prolongs glucose release without necessarily reducing total bioavailability. In summary, this study not only provides a new path for the development of healthy baked products using structured plant oils and microalgae, but also demonstrates the effectiveness of emulsion gel in improving the comprehensive quality of complex food systems, as evidenced by the enhanced sensory properties, modulated starch digestion kinetics, and improved fatty acid profile of the resulting cookies.

## Figures and Tables

**Table 1:** Amino acid composition of four microalgae (g/100g sample).

Name	Area			
	Chlorella Vulgaris	Dunaliella Salina	Nannochloropsis	Spirulina
Asp	2.152	1.262	1.771	2.198
Thr	0.983	0.537	0.829	0.925
Ser	0.780	0.474	0.662	0.927
Glu	2.890	1.501	2.064	3.178
Pro	1.21	0.91	2.83	1.42
Gly	1.313	0.574	0.949	0.885
Ala	1.771	0.666	1.135	1.309
Cys	0.052	0.034	0.045	0.038
Val	1.157	0.603	0.872	0.978
Met	0.290	0.145	0.198	0.213
Ile	0.668	0.380	0.553	0.824
Leu	1.456	0.741	0.996	1.172
Tyr	0.493	0.307	0.325	0.460
Phe	1.039	0.599	0.578	0.719
Lys	2.032	0.568	1.045	1.012
His	0.398	0.187	0.221	0.230
Arg	1.230	0.532	0.799	1.144
Total	18.70	9.11	13.04	16.21
NH^3+^	2.34	0.96	1.58	1.58

Note: According to GB 5009.124-2016, amino acid quantification was performed using single-point external standard calibration with certified amino acid reference standards; peak areas were integrated and converted to concentrations accordingly. Tryptophan was not determined due to its instability under standard acid hydrolysis conditions.

**Table 2:** Textural properties of *Nannochloropsis gaditana* cookies (mean ± SD, *n* = 6).

Sample	Hardness/g	Elasticity	Cohesiveness	Gumminess	Chewiness	Resilience
KB1	37,328.89 ± 0.13 a	0.40 ± 0.12 ab	0.30 ± 0.01 a	11,063.65 ± 0.11 a	4472.48 ± 0.15 a	0.21 ± 0.01 a
KB2	32,006.29 ± 0.09 b	0.49 ± 0.06 a	0.21 ± 0.02 b	6828.74 ± 0.09 b	3308.00 ± 0.11 ab	0.17 ± 0.01 b
WZ	28,315.00 ± 0.11 c	0.29 ± 0.06 b	0.21 ± 0.01 b	6066.95 ± 0.11 b	1765.53 ± 0.12 b	0.16 ± 0.01 b

Note: Different letters in the same row indicate significant differences (*p* < 0.05). Due to the absence of a gel-only control group, comparisons reflect the combined effects of the ingredients rather than isolated individual effects.

**Table 3:** Volatile flavor compounds identified in cookie formulations.

Types	Flavor Compounds	KB1	KB2	WZ
Pyrroles	2-Acetyl pyrrole	√	√	√
Pyrrolidines	1-Formylpyolidine	ND	√	√
	1-Acetylpyrrolidine	ND	√	√
Pyrazines	Pyrazine	√	ND	ND
	2-Methylpyrazine	√	√	√
	2,5-Dimethyl pyrazine	√	√	ND
	2,6-Dimethylpyrazine	√	ND	ND
	Ethylpyrazine	√	√	√
	2,3-Dimethylpyrazine	√	√	√
	2-Ethyl-6-methylpyrazine	√	√	√
	3-Ethyl-2,5-diMethylpyrazine	√	√	√
	2,3-Dimethyl-5-ethylpyrazine	√	ND	ND
	2-Ethyl-5-methylpyrazine	ND	ND	√
	2-Methyl-6-vinylpyrazine	ND	ND	√
	Acetylpyrazine	√	ND	ND
Alcohols	1-Hexanol	√	√	√
	1-Octen-3-ol	√	ND	√
	2,4-dimethylheptan-1-ol	√	ND	ND
	Trans-5-decen-1-ol	√	√	√
	1-Nonanol	√	ND	ND
	Furfuryl alcohol	√	√	√
	Benzyl alcohol	√	ND	ND
	Phenethyl alcohol	√	ND	ND
	Benzenemethanol, a-2-cyclohexen-1-yl-	ND	√	ND
Heterocyclic compounds	1-Methylazetidin-3-amine	ND	√	ND
Phenols	Guaiacol	√	ND	ND
Aldehydes	3-Methylbutanal	ND	√	√
	Hexanal	√	√	√
	Heptaldehyde	ND	ND	√
	Nonanal	√	√	√
	Benzaldehyde	√	√	√
	2-Pyridinecarboxaldehyde	ND	√	ND
	5-Methyl furfural	√	√	√
	2,6-Nonadienal	ND	√	ND
	2-Acetylthiazole	√	√	ND
	Pyrrole-2-carboxaldehyde	ND	ND	√
Acids	Acetic acid	ND	√	√
	Propionic acid	ND	√	√
	Butyric Acid	ND	√	ND
	Isovaleric acid	ND	√	√
	Hexanoic acid	√	√	√
	Octanoic acid	ND	√	√
	Nonanoic acid	ND	√	√
Ketones	Acetoin	√	ND	ND
	6-Methyl-5-hepten-2-one	√	√	ND
	1-(6-Methyl-2-Pyrazinyl)Ethanone	√	ND	ND
	3,5-Octadien-2-one	ND	√	ND
	2-Piperidone	ND	√	√
	3-Ethyl-4-methyl-pyrrole-2,5-dione	ND	√	√
	2,3-Dihydro-3,5-dihydroxy-6-methyl-4(H)-pyran-4-one	ND	√	√
	3,5-octadiene-2-one	ND	ND	√
	2(5H)-Furanone	ND	ND	√
	2-Pyrrolidinone	ND	ND	√
Esters	gamma-butyrolactone	√	ND	√
	4-Hexanolide	√	√	√
	5-Decanolide	√	√	ND
	Dihydroactinidiolide	ND	√	√

Note: ND = not detected; √ = detected. Compounds were identified by comparison of mass spectra with the NIST library and retention indices from the literature. This table presents qualitative presence/absence data only; relative abundances were not quantified.

**Table 4:** Fatty acid composition of different cookie formulations (g/100g sample).

Fatty Acids	Concentration		
	KB1	KB2	WZ
C6:0	ND	ND	0.051
C8:0	0.021	0.029	0.067
C10:0	0.016	0.022	0.016
C11:0	ND	ND	0.007
C12:0	0.111	0.151	0.069
C14:0	0.111	0.142	0.087
C15:0	0.007	0.009	0.011
C16:0	4.192	5.255	5.493
C16:1	0.061	0.094	0.100
C17:0	ND	ND	0.029
C18:0	0.564	0.685	0.686
C18:1n9	5.864	6.883	7.035
C18:2n6	8.096	8.768	8.679
C20:1	0.039	0.045	0.051
C18:3n3	0.100	0.103	0.083
C22:0	0.010	0.011	0.014
C23:0	ND	0.004	ND
C24:0	0.008	ND	ND
SFA	5.040	6.308	6.532
MUFA	5.964	7.022	7.185
PUFA	8.196	8.871	8.762

Note: KB1: control (no gel, no algae); KB2: algae only; WZ: algae + gel. ND: not detected.

## Data Availability

The original contributions presented in this study are included in the article. Further inquiries can be directed to the corresponding authors.
